# Gender-specific modulation of immune system complement gene expression in marine medaka *Oryzias melastigma* following dietary exposure of BDE-47

**DOI:** 10.1007/s11356-012-0887-z

**Published:** 2012-07-21

**Authors:** Roy R. Ye, Elva N. Y. Lei, Michael H. W. Lam, Alice K. Y. Chan, Jun Bo, Jason P. van de Merwe, Amy C. C. Fong, Michael M. S. Yang, J. S. Lee, Helmut E. Segner, Chris K. C. Wong, Rudolf S. S. Wu, Doris W. T. Au

**Affiliations:** 1 State Key Laboratory in Marine Pollution, Department of Biology and Chemistry, City University of Hong Kong, Kowloon, Hong Kong; 2 National Research Lab of Marine Molecular and Environmental Bioscience, Department of Chemistry College of Natural Sciences, Hanyang University, Seoul, South Korea; 3 Centre for Fish and Wildlife Health, University of Bern, CH3012 Bern, Switzerland; 4 Department of Biology, Baptist University of Hong Kong, Kowloon Tong, Hong Kong; 5 School of Biological Science, The University of Hong Kong, Pokfulam, Hong Kong

**Keywords:** PBDEs, BDE-47, Marine medaka, Immunomodulatory effects, Complement system, C3, Gender difference

## Abstract

BDE-47 is one of the most widely found congeners of PBDEs in marine environments. The potential immunomodulatory effects of BDE-47 on fish complement system were studied using the marine medaka *Oryzias melastigma* as a model fish. Three-month-old *O. melastigma* were subjected to short-term (5 days) and long-term (21 days) exposure to two concentrations of BDE-47 (low dose at 290 ± 172 ng/day; high dose at 580 ± 344 ng/day) via dietary uptake of BDE-47 encapsulated in *Artemia* nauplii. Body burdens of BDE-47 and other metabolic products were analyzed in the exposed and control fish. Only a small amount of debrominated product, BDE-28, was detected, while other metabolic products were all under detection limit. Transcriptional expression of six major complement system genes involved in complement activation: *C1r/s* (classical pathway), *MBL-2* (lectin pathway), *CFP* (alternative pathway), *F2* (coagulation pathway), *C3* (the central component of complement system), and *C9* (cell lysis) were quantified in the liver of marine medaka. Endogenous expression of all six complement system genes was found to be higher in males than in females (*p* < 0.05). Upon dietary exposure of marine medaka to BDE-47, expression of all six complement genes were downregulated in males at day 5 (or longer), whereas in females, MBl-2, CFP, and F2 mRNAs expression were upregulated, but C3 and C9 remained stable with exposure time and dose. A significant negative relationship was found between BDE-47 body burden and mRNA expression of C1r/s, CFP, and C3 in male fish (*r* = −0.8576 to −0.9447). The above findings on changes in complement gene expression patterns indicate the complement system may be compromised in male *O. melastigma* upon dietary exposure to BDE-47. Distinct gender difference in expression of six major complement system genes was evident in marine medaka under resting condition and dietary BDE-47 challenge. The immunomodulatory effects of BDE-47 on transcriptional expression of these complement components in marine medaka were likely induced by the parent compound instead of biotransformed products. Our results clearly demonstrate that future direction for fish immunotoxicology and risk assessment of immunosuppressive chemicals must include parallel evaluation for both genders.

**Electronic supplementary material:**

The online version of this article (doi:10.1007/s11356-012-0887-z) contains supplementary material, which is available to authorized users.

## Introduction

Polybrominated diphenyl ethers (PBDEs) have been commonly used as flame retardants in a wide range of commercial and household products in the past four decades (Siddiqi et al. 2003). Because of their usage and persistence, PBDEs are found ubiquitously in marine environments globally from the Antarctic, Scotland to Australia as well as in Asia including Korea and Hong Kong (Moon et al. 2007; Cheung et al. 2008; Hale et al. 2008; Webster et al. 2008; Toms et al. 2008). Among the many PBDE congeners, 2,2′,4,4′-tetra-bromodiphenyl ether (BDE-47) predominates in marine biota, comprising over 50 % of the total PBDEs detected in marine fish (Akutsu et al. 2001; Boon et al. 2002; Brown et al. 2006; Meng et al. 2008; She et al. 2002; Voorspoels et al. 2003). Given the wide distribution and bioaccumulation potential of BDE-47 in fish, considerable concern has been raised about its potential effects on health and fitness of contaminated fish. A recent study using juvenile Chinook salmon showed that PBDEs decreased host resistance against pathogenic bacteria *L. anguillarum* (Arkoosh et al. 2010).

**Fig. 1:**
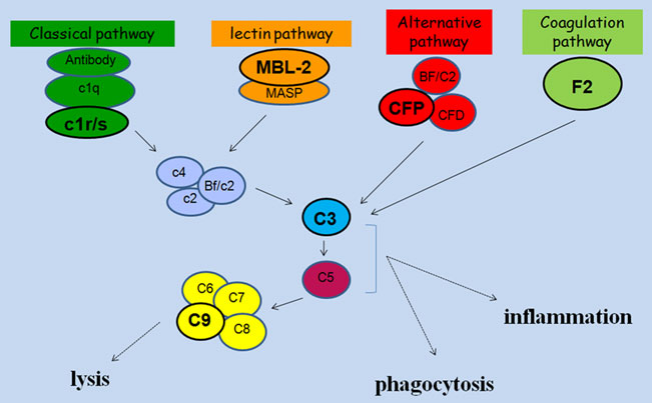
Hypothetical complement activation pathways in marine medaka *Oryzias melastigma*, leading to inflammation, phagocytosis, and cell lysis (modified from Gonzalez et al. 2007; Amara et al 2008). Four complement genes of marine medaka, representing the four activation pathways: *C1r/s* (classical pathway), *MBL-2* (lectin pathway), *CFP* (alternative pathway), and *F2* (coagulation pathway) as well as the complement C3 (central protein of all four activation pathways) and complement C9 (cell lysis) were chosen for the present study

In fish, the complement system serves as the first line of host defense against pathogens and bridges innate and adaptive immunity (Carroll 2004; Ricklin et al. 2010). In vertebrates, the liver is the major site of complement component synthesis and four complement activation pathways (classical, lectin, alternative, and coagulation pathways) have been identified (Holland and Lambris 2002; Boshra et al. 2006) (Fig. 1). The classical pathway is activated by interaction between C1 complex (comprising C1q, C1r, and C1s) with antibody, which in turn activates C4 and C2, leading to activation of C3 (Ricklin et al. 2010). In the lectin activation pathway, binding of mannose-binding lectin-2 (MBL-2) onto microbial surface initiates autoactivation of MBL-associated serine proteases (MASPs), cleavage of C4 and C2, and activation of C3 (Ip et al. 2009). In the alternative pathway, complement factor properdin (CFP) is critical in spontaneous activation of C3 (or “tick over”) (Kemper and Hourcade 2008). The fourth activation pathway has been discovered recently, which is activated by coagulation factor II (F2), a critical protein in the coagulation system (Amara et al. 2008; Huber-Lang et al. 2006). Complement component C3 is the central protein of all known complement activation pathways (Boshra et al. 2006; Holland and Lambris 2002). Activation of C3 will lead to downstream activation of C5 to C9, promoting phagocytosis, local inflammation, and formation of membrane attack complex (MAC) (Boshra et al. 2006). C9 is the final component of MAC, and polymerization of C9 can form a transmembrane channel to destroy the phospholipid bilayer of target cells, resulting in cell lysis (Ricklin et al. 2010).

*Oryzias melastigma* has been shown to be a promising marine fish model for ecotoxicological and innate immune studies (Bo et al. 2011a, Kong et al. 2008; Shen et al. 2010). The complement genes *MBL-2*, *CFP*, *F2*, and *C3* have been identified from our suppression subtractive hybridization (SSH) library of *O. melastigma* liver (Bo et al. 2011b). In both the marine medaka *O. melastigma* and the Japanese medaka *Oryzias latipes*, two gene sequences with high homology to C9 as well as to C1r and C1s, namely C1r/s, were found. The complement system is highly conserved in vertebrates. The hypothetical complement activation pathways in marine medaka are shown in Fig. 1.

It has long been proposed that PBDEs are endocrine-disrupting compounds (Costa et al. 2008; Legler and Brouwer 2003; Rahman et al. 2001). Complement component mRNA expression, for instance, C1q, C3, and C7 of juvenile/male fish, is highly responsive to estrogen-active chemicals (see review of Casanova-Nakayama et al 2011). Furthermore, larvae of Japanese medaka (*O. latipes*) exposed to 17β-estradiol (0.1–10 μg/L for 30 days) showed a concentration- and time-dependent suppression in mRNA expression of three complement components (C3-1, C3-2, and Bf/C2) (Sun et al. 2011). Similar analyses were not conducted for mature female fish with a high endogenous level of estrogen. Our earlier study demonstrated for the first time and gender difference in time of mRNAs induction and extent of elevation of hepcidin, an antimicrobial protein, in immune organs (liver and spleen) of marine medaka *O. melastigma* upon bacterial challenge (F > M) (Bo et al. 2011a). It is possible that the complement system genes of *O. melastigma* may also respond to PBDEs in a gender-specific manner. Given the high bioaccumulation potential of BDE-47, the present study aimed to test the hypothesis that expression of major complement genes involved in activation of the complement system (including four key pathway genes *C1r/s*, *MBL-2*, *CFP*, and *F2* as well as the central C3 and downstream C9 component genes) in male and female marine medaka can be differentially modulated by BDE-47 via dietary uptake (*Artemia* nauplii). The results of this study provide vital evidence to substantiate that gender-specific response is prominent in fish, which should be taken into consideration for risk assessment of immunomodulatory chemicals.

## Materials and methods

### Bioencapsulation of BDE-47 in *Artemia* nauplii

Methodology for bioencapsulation of BDE-47 in *Artemia* followed the methods described in our earlier study (van de Merwe et al. 2011) with minor modification. Briefly, stock solution of BDE-47 (98.5 % purity; ChemService, Inc., USA) was prepared in hexane to 10 mg/mL concentration (working solution). A working solution of BDE-47 (825 μL) of 10 mg/mL was added to a 150-mL conical flask. The hexane was evaporated and 100 mL of newly hatched *Artemia* nauplii (~1,500 nauplii/mL, 3.87 ± 2.3 BDE-47 ng/nauplii) was added to the BDE-47 deposited and hexane-free flask. After incubation for 24 h, *Artemia* nauplii were harvested, rinsed thoroughly, resuspended in Milli-Q water (to ~1,500 *Artemia* nauplii/mL) and stored at −20 °C. Bioencapsulation of BDE-47 following these procedures produced *Artemia* nauplii that when fed daily to 3-month-old marine medaka resulted in environmentally relevant BDE-47 concentrations for fish (see van de Merwe et al. 2011). A separate batch of *Artemia* nauplii was prepared in the same way as the control (adding hexane only without BDE-47 to flask).

### Dietary exposure of marine medaka to BDE-47

The marine medaka *O. melastigma* used in this experiment were from stock originally purchased from Interocean Industries (Taiwan) and reared in the State Key Laboratory in Marine Pollution, City University of Hong Kong for more than 30 generations. Each glass tank (15 cm × 15 cm × 15 cm), with a removable glass divider, was used to accommodate one 3-month-old female and one 3-month-old male marine medaka. Each glass tank was filled with 2 L of 30 ‰ artificial sea water and kept in stable environment (22 ± 1 °C) on a 14:10 h of light:dark cycle with gentle aeration. Half of the water was changed and waste removed every other day. Fish were randomly assigned to control, low dose, or high dose treatment groups. To ensure that each fish consumed the same amount of *Artemia* nauplii over the course of the experiment, fish were separated in each tank with the glass dividers immediately prior to feeding. Each day, individual fish were fed either 100 μL of uncontaminated *Artemia* nauplii (controls), 100 μL of BDE-47 bioencapsulated *Artemia* nauplii (high-dose exposure), or 50 μL of each (low-dose exposure). The glass dividers were removed after all the *Artemia* nauplii were consumed by each fish (approximately 15 minutes). All fish were also fed hormone-free flake fish food to satiation (AX5; Aquatic Ecosystems, USA) twice per day to maintain growth and condition. At day 5 and day 21, fish in the BDE-47 treatment groups and the control group were sampled and freeze-dried for BDE-47 body burden analysis (*n* = 5 for each sex and treatment), and liver of fish were isolated and stored in −80 °C for quantitative analysis of complement system gene expression by real-time PCR (*n* = 10 for each sex in the low-dose/control group; *n* = 6 for each sex in the high-dose group).

### Test chemicals

All chemicals used were purchased from Sigma-Aldrich (St. Louis, MO), except the following: PBDE congeners and ^13^C-labeled PBDE congeners (Wellington Laboratories, Guelph, Ontario, Canada), methoxy and hydroxy metabolites of PBDE congeners (MeO-PBDEs and OH-PBDEs, respectively) (in-house synthesis).

### Bioaccumulation of BDE-47

Freeze-dried carcass samples (ca. 8 mg) were grounded with 1.5 g of anhydrous sodium sulfate and spiked with 50 ppb of 10-μL ^13^ C-labeled BDE-47 surrogates for 3 days prior to sample preparation. The sample extraction and workup procedures were modified from Wan et al. (2010). Prepared samples were extracted by two rounds of accelerated solvent extraction (10 min for each round) by *n*-hexane and dichloromethane (DCM) (1:1 ^v^/_v_) at 100 °C, followed by *n*-hexane and methyl butyl ether (MTBE) (1:1 ^v^/_v_) at 60 °C, using a Dionex ASE-350 accelerated solvent extractor (Sunnyvale, CA). Lipid content of the samples was determined by microcolorimetric sulfo-phospho-vanillin (SPV) analysis modified from Lu et al. (2008).

The extract was treated with a 50 % ethanolic potassium hydroxide solution. The “neutral fraction” of the extract was obtained by directly extracting the resulting aqueous layer with *n*-hexane. The “phenolic fraction” was obtained by extraction of the aqueous layer, after its acidification by 2.0 M hydrochloric acid, *n*-hexane/MTBE (9:1 ^v^/_v_). Cleanup of these fractions were carried out by column chromatography (from top to bottom: 250 mg of anhydrous sodium sulfate, 400 mg of acidified silica, 400 mg of neutral alumina, and 300 mg anhydrous sodium sulfate). The “neutral fraction” was eluted by *n*-hexane/DCM (1:1 ^v^/_v_) and reconstituted in *n*-hexane for GC–MS analysis. Quantification of the various PBDE congeners and methoxy metabolites were conducted via standard addition. A 50 ppb of 10-μL ^13^ C-labeled BDE-77 and BDE-138 were used as internal standards.

The “phenolic fraction” was derivatized by ethyl chloroformate (ECF) in a solvent mixture of acetonitrile, methanol, water, and pyridine in 5:2:2:1 ratio (v/v/v/v) at room temperature for 1 h, following by quenching in Milli-Q water and extraction with *n*-hexane before cleanup by column chromatography. The “phenolic fraction” was eluted by *n*-hexane/DCM (1:1 ^v^/_v_) and reconstituted in *n*-hexane for GC–MS analysis.

Qualitative and quantitative determination of all target compounds was performed using an Agilent Technologies 7890A GC, with a split/splitless injector, interfaced to a 5975C inert XL EI/CI mass spectrometer (GC–MSD). Chromatographic separation was achieved on a DB5-MS fused silica capillary column (30 m length, 0.25 mm ID, 0.1 μm film thickness, Agilent, Carlsbad, CA). Helium was used as the carrier gas. The GC temperature program is shown in Table S1 of the supporting information. The mass spectrometer was operated in the Selected Ion Monitoring (SIM) mode. Ions monitored for PBDEs, MeO-PBDEs, ECF derivatized OH-PBDEs, and BRP in the chromatogram are shown in Tables S2 and S3 of the supporting information. The method detection limits for the various brominated compounds are shown in Table 1

**Table 1:** Quality assurance parameters for PBDEs and potential biotransformation products

BDE congeners	Spiked recovery (2 ng) (*n* = 12)	Spiked recovery (100 ng) (*n* = 12)	Relative repeatability	Method detection limit (μg per kg lipid)
BDE-3	64.600 % ± 26.517 %	n/a	10.0225 %	10.5
BDE-15	77.829 % ± 42.996 %	n/a	16.2510 %	6.6
BDE-28	79.900 % ± 26.428 %	n/a	9.9888 %	10.5
BDE-47	77.303 % ± 14.766 %	n/a	5.5810 %	5.9
5-OMe BDE-47	76.506 % ± 16.716 %	n/a	6.3181 %	6.6
6-OMe BDE-47	80.589 % ± 21.011 %	n/a	6.0654 %	8.3
3-ECFO BDE-47	n/a	77.821 % ± 11.381 %	3.2854 %	1.8
6-ECFO BDE-47	n/a	88.219 % ± 12.240 %	3.5334 %	1.9

PBDE congeners and ^13^C-labeled PBDE congeners was purchased from Wellington Laboratories (Guelph, Ontario, Canada); purities of PBDEs and ^13^C-labeled PBDEs were 100 ± 5 %. Methoxy and hydroxyl metabolites of PBDE congeners (MeO-PBDEs and OH-PBDEs, respectively) was synthesized in the Department of Biology and Chemistry of City University of Hong Kong following the methods described by Marsh et al. (2003); purities of all metabolites were >98 %

### Marine medaka gene sequences

The gene sequence for complement component MBL-2, CFP, F2, and C3 were obtained from the SSH cDNA library for *O. melastigma* (Bo et al. 2011b), annotated by Gene Ontology (http://www.geneontology.org/). A C3 partial sequence was identified with equal similarity to the two Japanese medaka C3 genes (*C3-1* and *C3-2*). Therefore, a C3 primer was designed based on these two sequences and that represents the total expression of different paralogue C3 in marine medaka. Two C1r- or C1s-like (ENSORLT00000015464, ENSORLT00000015483) genes are predicted by Ensemble Japanese medaka database. Due to a higher similarity to the reported fish C1r/s amino acid sequences (supporting information Table S4), the ENSORLT00000015464 was selected to blast against by the marine medaka genome database (JS Lee, Hanyang University), and the sequence was named C1r/s based on phylogenetic analysis (Fig. S1 in supporting information). One C9-like gene (ENSORLT00000003702) is predicted in Ensemble and used for blasting against marine medaka genome database. Reference of marine medaka gene sequence can be found in NCBI nucleotide database, C1r/s (JQ045127), MBL-2 (HM137110), CFP (HQ144250), F2 (HM137108), C3 (HM137119), and C9 (JQ045126).

### Real-time PCR for complement gene expression

Fish liver was homogenized by sterilized micropestles, total RNA extracted by TRIzol Reagent (Invitrogen, Hong Kong), and cDNA was synthesized using One-Step TaKaRa Primescript™ RT Reagent Kit (TaKaRa, China). Briefly, the assay was performed using Power SYBR Green PCR Master Mix (ABI, Hong Kong) in ABI 7500 System. Gene-specific primers were designed by Beacon Designer. The primer sequences for the six complement component genes and the reference gene are listed in Table 2. Relative mRNA expression level was calculated by the classical 2^−ΔΔCT^ method using 18 s rRNA as endogenous control gene.

**Table 2:** Primers used for real-time PCR measurement

Gene name	Primer sequence (forward and reverse) (5′ → 3′)	Product length (bp)	Primer efficiency
*C1r/s*	F:TGTCCAGTCCAGGCTATC	132	103.1 %
	R:GTTGAGAGTATTGACACAGAGG		
*MBL-2*	F: CTGCAGCTTTGCCGCCATCG	113	99.6 %
	R: GCAGCTGGCAGTGCTCCACA		
*CFP*	F:AACCCACATTATGGCTACCTG	128	105.8 %
	R:CGATGGCTCTGCCTCACTC		
*F2*	F:CAGCAGTCCAGAAAGAAA	239	96.4 %
	R: CTTGTTGCCAATCAGTTG		
*C3*	F: GGTCAAGAGTGAATGGAATGCCTA	176	100.3 %
	R: CTAACAGAAACAAGATGGAGAGCC		
*C9*	F:AAGACAATGATACAGGATAAGAC	80	102.2 %
	R:GACATAGACGGCTCAGAT		
*18S*	F: CCTGCGGCTTAATTTGACCC	134	100.5 %
	R: GACAAATCGCTCCACCAACT		

### Statistical analysis

BDE-47 concentration in marine medaka was normalized with lipid weight before data analysis. An independent *t* test was used to test the null hypothesis, and no gender difference was found in endogenous expression of each complement gene in marine medaka (*p* < 0.05). Differences in normalized BDE-47/gene expression levels between different doses and exposure periods in male/female fish were analyzed by two-way ANOVA followed by Tukey's post hoc test. Pearson's correlation analysis was performed for mean BDE-47 body burden (log transformed) and mean mRNA level for each complement gene in fish (*n* = 6 from two exposure times × three treatment groups). Statistical analyses were carried out using SigmaStat 3.5 and Prism 5.

## Results

### BDE-47 in *Artemia* nauplii

The average concentration of BDE-47 in the *Artemia* nauplii was 3.87 ± 2.3 ng/nauplii. The amount of BDE-47 fed to each fish per day (estimated from the average concentration of BDE-47 in the *Artemia* nauplii multiplied by the mean number of *Atermia* nauplii fed to each fish) was found to be 290.3 ± 172.3 ng/day in the low-dose group and 580.5 ± 344.6 ng/day in the high dose group. No other BDE congeners, OMe-BDEs, and OH-BDEs were detectable in the *Artemia* nauplii.

### BDE-47 and its metabolomic products in medaka

**Fig. 2:**
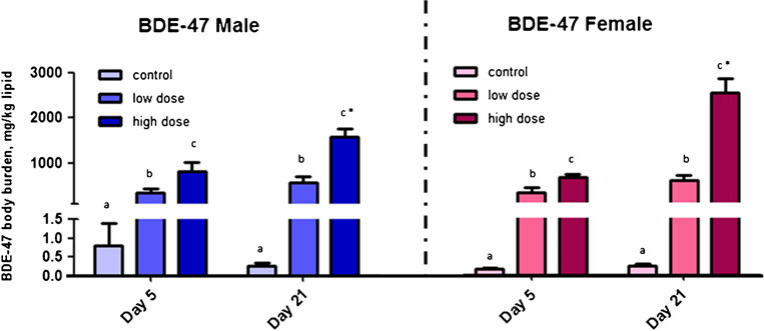
Body burden of BDE-47 in male (*blue*) and female (*red*) in *Oryzias melastigma* (mean ± standard error, *n* = 5) after exposure to low dose (290.3 ng/day) and high dose (580.5 ng/day) BDE-47 for 5 days and 21 days. *Different letters* indicate significant difference between treatments within the same day (*p* < 0.05). *Asterisk* indicates difference between time within the same treatment (*p* < 0.05); two-way-ANOVA and Tukey's post hoc test

At day 5, BDE-47 in the low-dose group was found to be 349.2 ± 214.0 mg/kg lipid in males and 325.3 ± 240.8 mg/kg lipid in females. In the high-dose group, BDE-47 in male and female medaka was 831.3 ± 433.4 mg/kg lipid and 670.0 ± 124.2 mg/kg lipid, respectively. In the control groups, BDE-47 was 0.23 ± 0.05 mg/kg lipid in males and 0.15 ± 0.07 mg/kg lipid in females (Fig. 2). BDE-28 in the low-dose group was found to be 1.003 ± 0.912 mg/kg lipid and 1.178 ± 0.877 mg/kg lipid in male and female medaka, respectively. In the high-dose group, BDE-28 was 1.134 ± 0.539 mg/kg lipid in males and 0.857 ± 0.371 mg/kg lipid in females.

At day 21, BDE-47 in the low-dose group was 576.4 ± 300.4 mg/kg lipid in males and 602.6 ± 702.3 mg/kg lipid in females. In the high-dose group, BDE-47 in male and female medaka was 1,547.9 ± 422.6 mg/kg lipid and 2,515.8 ± 702.3 mg/kg lipid, respectively. In the control groups, BDE-47 was 0.25 ± 0.2 mg/kg lipid in males and 0.24 ± 0.1 mg/kg lipid in females (Fig. 2). BDE-28 in the low-dose group was 0.809 ± 0.555 mg/kg lipid in males and 0.713 ± 0.208 mg/kg lipid in females, respectively. In the high-dose group, BDE-28 in male and female medaka was 1.240 ± 0.529 mg/kg lipid and 2.211 ± 0.943 mg/kg lipid, respectively. Besides BDE-47 and BDE-28, other BDE congeners, OMe-BDEs, and OH-BDEs were not detected either at day 5 or day 21.

### Gender difference in expression of complement system genes in marine medaka

Results of the independent *t* test show a clear gender difference in endogenous mRNA level of each of the six complement system genes used in the present study, in which the males ubiquitously exhibit a significantly higher mRNA level than the female fish (Table 3).

**Table 3:** Gender difference in endogenous expression level of six complement genes in marine medaka *Oryzias melastigma*

	*C1R/S*	*MBL-2*	*CFP*	*F2*	*C3*	*C9*
Day 5	M > F^**^	M > F^***^	M > F^*^	M > F^**^	M > F^**^	M > F^***^
Day 21	M > F^***^	M > F^*^	M > F^***^	M > F^***^	M > F^***^	M > F^***^

Control fish at day 5 and day 21 used in the BDE-47 *Artemia* nauplii feeding experiment were compared

*M* male fish, *F* female fish

^*^ *p* < 0.05 (significant difference between genders, unpaired *t* test)

^**^ *p* < 0.01 (significant difference between genders, unpaired *t* test)

^***^ *p* < 0.001 (significant difference between genders, unpaired *t* test)

### The effects of BDE-47 on transcription of complement system genes

**Fig. 3:**
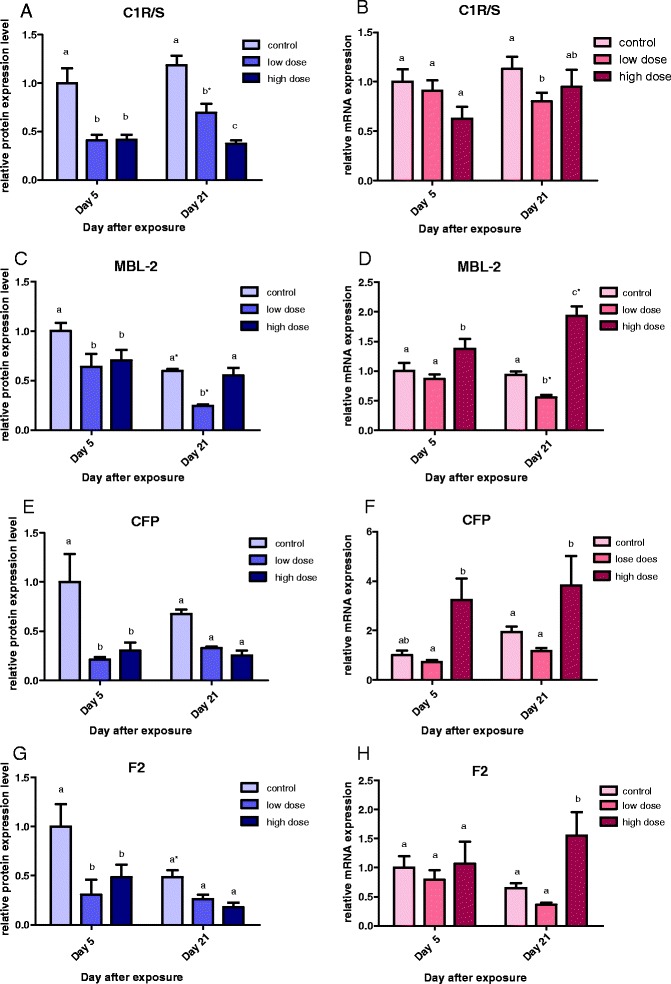
Relative mRNA expression level of four complement genes: *C1r/s* (**a** and **b**), *MBL-2* (**c** and **d**), *CFP* (**e** and **f**), and *F2* (**g** and **h**) in male (*blue*) and female (*red*) *Oryzias melastigma* after exposure to low dose (290.3 ng/day) and high dose (580.5 ng/day) BDE-47 for 5 and 21 days. Transcription levels were normalized to 18 S rRNA levels and expression level of the day 5 control was adjusted to 1 for relative comparison among different times and treatments (mean ± standard error, *n* = 10 for the control/low dose groups, *n* = 6 for the high dose group). *Different letters* within indicate significant difference between treatments in the same day (*p* < 0.05); *asterisk* indicates difference between time within the same treatment (*p* < 0.05)

For C1r/s (classical pathway) expression in males, C1r/s mRNA was downregulated in both high-dose and low-dose BDE-47 at day 5 (*p* < 0.05) (Fig. 3a). A clear dose-dependent suppression of C1r/s mRNA expression was evident at day 21 (*p* < 0.05) (Fig. 3a). In females, no significant difference was found between the control and treatment groups, except a reduced C1r/s mRNA level in the low-dose group at day 21 (Fig. 3b).

For MBL-2 (lectin pathway) expression in males, MBL-2 mRNA was downregulated in both low- and high-dose BDE-47 treatments (*p* < 0.05), except in the high-dose group at day 21, in which the level in the control group was also reduced (Fig. 3c). In females, MBL-2 transcript level was reduced in the low-dose group at day 21. Conversely, a time-dependent increase in expression of MBL-2 mRNA was found in the high-dose group (*p* < 0.05) (Fig. 3d).

For CFP (alternative pathway) expression in males, CFP mRNA was significantly downregulated in both BDE-47 treatments at day 5 only (*p* < 0.05), but not at day 21 (Fig. 3e). Conversely, in females, CFP transcript level was elevated in the high-dose group at day 21 (*p* < 0.05), whereas no significant change was found in the low-dose group at both times (Fig. 3f).

For F2 thrombin (coagulation pathway) expression in male fish, F2 mRNA was significantly downregulated in both treatment groups at day 5 and the transcript level in the control was also reduced in day 21 compared to day 5 (*p* < 0.05) (Fig. 3g). In female fish, F2 mRNA expression was upregulated in the high-dose group at day 21 (*p* < 0.05), whereas no significant difference was found between the control and low-dose group at both times (Fig. 3h).

**Fig. 4:**
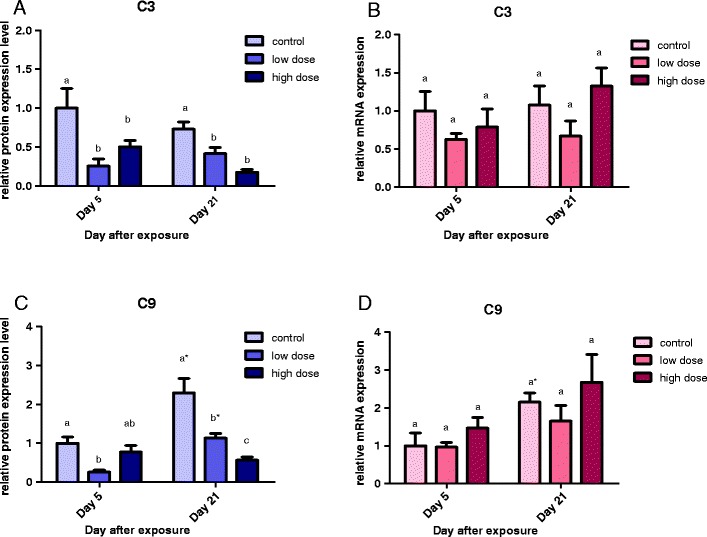
Relative mRNA expression levels of complement genes: *C3* (**a** and **b**) and *C9* (**c** and **d**) in male (*blue*) and female (*red*) *Oryzias melastigma* after exposure to low dose (290.3 ng/day) and high dose (580.5 ng/day) of BDE-47 for 5 and 21 days. Transcription levels were normalized to 18 S rRNA levels and expression level of the day 5 control was adjusted to 1 for relative comparison among different times and treatments (mean ± standard error, *n* = 10 for the control/low dose groups, *n* = 6 for the high dose group). *Different letters* indicate significant difference between treatments in the same day (*p* < 0.05); *asterisk* indicates difference between time within the same treatment (*p* < 0.05)

C3 is the central protein of the complement system (Fig. 1). In male fish, C3 mRNA expression was downregulated in both BDE-47 treatments at day 5 and day 21 (*p* < 0.05) (Fig. 4a). However, in female fish, there was no significant difference in C3 transcript level between treatments and control at both times (Fig. 4b).

C9 is a downstream complement component leading to cell lysis. In male fish, reduced C9 mRNA expression was found in the low-dose and high-dose groups at different times (Fig. 4c). In female fish, there was no significant change in C9 transcript level in both treatments over time, except that the level in the control group was elevated at day 21 as compared to day 5 (Fig. 4d).

### Correlation between BDE-47 body burden and complement gene expression

The relationship of BDE-47 body burden in fish and expression of each complement system genes was examined separately in male and female marine medaka at day 5 and day 21. In males, a significant negative correlation was found for C1r/s, CFP, and C3 (Pearson's *r* = −0.9447, −0.8881, and −0.8576, respectively) (*p* < 0.05) (Table 4). However, in female fish, no significant correlation was found between BDE-47 body burden and any gene expression level.

**Table 4:** A summary of correlation coefficient of BDE-47 body burden and complement component gene expressions in the liver

	*C1R/S*	*MBL-2*	*CFP*	*F2*	*C3*	*C9*
Male	−0.9447^*^	−0.4946	−0.8881^*^	−0.7114	−0.8576^*^	−0.7432
Female	−0.6782	0.3473	0.4349	0.249	−0.2129	0.2334

^*^ *p* < 0.05

## Discussion

### BDE-47 and its metabolic products in fish

After 5 days of exposure to BDE-47, its bioaccumulation in marine medaka was ca. 28.8 % of the estimated ingested amount in males and 30.1 % in females. The level increased to 64.0 % in males and 52.8 % in females, after an additional 16 days of dietary exposure. Body burden of BDE-47 in the low-dose group and high-dose group of marine medaka reached 300–900 mg BDE-47/kg lipid and 1,500–2,500 mg BDE-47/kg lipid in low-dose and high-dose fish, respectively.

Only ca. 0.07–0.10 % of the ingested BDE-47 was debrominated to BDE-28 in marine medaka during the study period. All other potential BDE-47 metabolites were below our method detection limits (Table 1). Our results show that BDE-47 was poorly metabolized in marine medaka, and similar results were also reported in Japanese medaka (Wan et al. 2010). These findings indicate that the effects on complement gene expressions in marine medaka upon exposure to the BDE congeners were likely induced by the parent compound instead of its biotransformed metabolic products. However, the possibility of very potent metabolite(s) causing the change, but not BDE-47, cannot be excluded.

### Gender difference of complement gene expression

The present study demonstrates for the first time distinct gender difference in endogenous expression of complement C1r/s, MBL-2, CFP, F2, C3, and C9 transcripts in *O. melastigma* (males > females). We further found that the responses of these complement genes in marine medaka upon exposure to BDE-47 are very different between males and females. Transcription of all six major complement system genes: *C1r/s* (an important serine protease in the classical activation pathway), *MBL-2* (plays a critical role in initiating the lectin activation pathway), *CFP* (promotes activation of the alternative pathway), *F2* (activates the coagulation pathway), *C3* (the central component of the complement system), and *C9* (a downstream component involving in the formation of MAC and cell lysis) were all downregulated in BDE-47 exposed males at day 5 and beyond (Figs. 3 and 4). In contrast, complement gene transcription was mostly upregulated (MBL-2, CFP, F2) or remained unchanged (C3, C9) in BDE-47 exposed females (Figs. 3 and 4). The results suggest that sexual dimorphism of complement system function may occur in fish, and gender-specific responses should be taken into consideration when assessing the risk of environmental contaminants on immune competence of fish.

We also found a significant negative correlation between BDE-47 body burden and mRNA expression of C1r/s/CFP/C3 in male fish (Table 3). The findings indicate that the classical complement activation pathway (C1r/s, C3) and alternative complement activation pathway (CFP, C3) in male *O. melastigma* are particularly sensitive to BDE-47 exposure. It is uncertain if upregulation of MBL-2, CFP, and F2 mRNAs, but an absence of change in C3 mRNA, in BDE-47 exposed females may lead to an enhancement of immune competence. However, an overall downregulation of all six major complement system genes in BDE-47 exposed males, in particular *C3*, is likely to impair fish complement system function and immune competence.

In mammals, C3 deficiency is associated with immunodeficiency and various immune diseases (Mueller-Ortiz et al. 2004; Reis et al. 2006). A recent study in fish has shown that a reduced upregulation of C3 gene expression in juvenile rainbow trout was associated with an increase of fish mortality after bacteria challenge (Casanova-Nakayama et al. 2011, Wenger et al. 2011). It is possible that the observed downregulation of complement genes (in particular C3) in BDE-47 treated male *O. melastigma* may impair complement system function. The adverse outcome pathways on C3 suppression and immune function impairments (e.g., using pathogens challenge experiments) will be further investigated in marine medaka.

BDE-47-mediated downregulation of complement component gene transcription may be related to its estrogenic effect. It has long been known that BDE-47 is a weak agonist/antagonist on estrogen receptors (Hamers et al. 2006; Liu et al. 2011; Meerts et al. 2001; Villeneuve et al. 2002). The estrogenic effect of BDE-47 is mediated through estradiol sulfotransferase. BDE-47 has been shown to inhibit sulfonation of E2 (IC50 = 0.8 μM, which was approximately threefold higher than the positive control, pentachlorophenol) (Hamers et al. 2006). Inhibition of E2 sulfonation will sustain the level of bioactive estradiol and lead to a prolonged estrogenic effect (Fisher 2004). A number of recent studies have shown that an estrogen-active compound, e.g., E2, is able to downregulate complement system and C3 transcription in juvenile/male fish (Williams et al. 2007; Sun et al. 2011; Wenger et al. 2011). The suppression effect of BDE-47 on C3 and complement gene transcription in BDE-47 exposed male *O. melastigma* could be attributed to an increase of estrogenic effect induced by BDE-47. Females with a high endogenous E2 level may not be as sensitive as males to BDE-47-induced estrogenic effect and, therefore, similar modulating effects would not occur in females upon the same BDE treatment.

## Conclusion

Results of this study provide clear evidence that endogenous expression of all six major complement system genes studied was gender-dependent (males > females). BDE-47 is not biotransformed in marine medaka. The immunomodulatory effects of BDE-47 on complement gene transcription were very different in male and female *O. melastigma*, suggesting gender-dependent response is an important consideration when assessing the risk of immunosuppressive chemicals. Future direction for fish immunotoxicology must include parallel assessment for both genders.

## Electronic supplementary material

Below is the link to the electronic supplementary material.

ESM 1 (DOCX 238 kb)
