# Supplementary material for: Gender-specific modulation of immune system complement gene expression in marine medaka Oryzias melastigma following dietary exposure of BDE-47
Source: Environ Sci Pollut Res Int. 2012 Jul 21;19(7):2477–87. doi: 10.1007/s11356-012-0887-z (PMC3404281; doi:10.1007/s11356-012-0887-z)
Supplement: Supplementary file 1 — (DOCX 238 kb) [file 11356_2012_887_MOESM1_ESM.docx]

Table S1. GC temperature program for GC-MSD of PBDEs, MeO-PBDEs, ECFO-PBDEs and ECFO-BRP.

| *Injector information* | |
| --- | --- |
| Mode | Pulsed splitless |
| Heater | 280°C |
| Pressure | 10.42psi |
| Total flow | 54.2mL/min |
| Septum purge flow | 3mL/min |
| Gas saver | 20mL/min |
| Injection pulse pressure | 20psi until 0.5min |
| Purge flow to split vent | 50mL/min at 2min |
| *Column information* | |
| Column | DB-5MS  with 30m length, 0.25mm ID, 0.1μm film thickness |
| Flow rate | Constant flow at 1.2mL/min |
| *Oven information* | |
| Equilibration time | 0.5min |
| Oven program | 60°C for 2min  then 15°C/min to 250°C for 0min  then 5°C/min to 280°C for 5min  then 30°C/min to 290°C for 20min |
| *Thermal Aux (MSD transfer line) information* | |
| Heater | 290°C |

Table S2. Ion monitored for GCMS of PBDEs and MeO-PBDEs in tissue extracts.

| Group no.  (Start time, min) | R*t*, min | *m/z*  (dwell time, msec) | *m/z* type | *m/z* formula | Substance |
| --- | --- | --- | --- | --- | --- |
| 1  (11.5) | 12.31 | 248 (200)  250 (200) | M  M + 2 | ^12^C_12_H_9_^79^BrO  ^12^C_12_H_9_^79^Br^81^BrO | BDE-3  (4-MoBDE) |
| 2  (14.3) | 14.50 | 326 (200)  328 (200)  330 (200) | M  M + 2  M + 4 | ^12^C_12_H_8_^79^Br_2_O  ^12^C_12_H_8_^79^Br^81^BrO  ^12^C_12_H_8_^81^Br_2_O | BDE-15  (4, 4’-DiBDE) |
| 3  (15.8) | 16.20 | 404 (200)  406 (200)  408 (200) | M  M + 2  M + 4 | ^12^C_12_H_7_^79^Br_3_O  ^12^C_12_H_7_^79^Br_2_^81^BrO  ^12^C_12_H_7_^79^Br^81^Br_2_O | BDE-28  (2, 4, 4’-TrBDE) |
| 4  (17.5) | 18.29 | 484 (150)  486 (150)  488 (150) | M  M + 2  M + 4 | ^12^C_12_H_6_^79^Br_4_O  ^12^C_12_H_6_^79^Br_3_^81^BrO  ^12^C_12_H_6_^79^Br_2_^81^Br_2_O | BDE-47  (2, 2’, 4, 4’-TeBDE) |
|  | 18.29 | 496 (100)  498 (100)  500 (100) | M  M + 2  M + 4 | ^13^C_12_H_6_^79^Br_4_O  ^13^C_12_H_6_^79^Br_3_^81^BrO  ^13^C_12_H_6_^79^Br_2_^81^Br_2_O | ^13^C-BDE-47 |
|  | 19.08 | 496 (100)  498 (100)  500 (100) | M  M + 2  M + 4 | ^13^C_12_H_6_^79^Br_3_O  ^13^C_12_H_6_^79^Br_3_^81^BrO  ^13^C_12_H_6_^79^Br_2_^81^Br_2_O | ^13^C-BDE-77 |
| 5  (19.2) | 19.58 | 514 (200)  516 (200)  518 (200) | M + 2  M + 4  M + 6 | ^12^C_13_H_8_^79^Br_3_^81^BrO_2_  ^12^C_13_H_8_^79^Br_3_^81^Br_2_O_2_  ^12^C_13_H_8_^79^Br^81^Br_3_O_2_ | 6-OMe  -BDE-47 |
| 6  (19.9) | 20.15 | 341 (200)  356 (200)  516 (200) | M-CH_3_^79^Br^81^Br  M - ^79^Br^81^Br  M + 2 | ^12^C_12_H_5_^79^Br_2_O_2_  ^12^C_13_H_8_^79^Br_2_O_2_  ^12^C_13_H_8_^79^Br_3_^81^BrO_2_ | 5-OMe  -BDE-47 |
| 7  (25.0) | 25.80 | 654 (200)  656 (200)  658 (200) | M + 4  M + 6  M + 8 | ^13^C_12_H_4_^79^Br_4_^81^Br_2_O  ^13^C_12_H_4_^79^Br_3_^81^Br_3_O  ^13^C_12_H_4_^79^Br_2_^81^Br_4_O | ^13^C-BDE-138 |

Table S3. Ion monitored for GCMS of ECFO-BPR and ECFO-PBDEs in tissue extracts.

| Group no.  (Start time, min) | R*t*, min | *m/z*  (dwell time, msec) | *m/z* type | *m/z* formula | Substance |
| --- | --- | --- | --- | --- | --- |
| 1  (12.0) | 13.78 | 249 (100)  251 (200)  253 (100)  266 (200) | M - CO_2_CH_3_  M - CO_2_CH_3_ + 2  M - CO_2_CH_3_ + 4  M - CO_2_ + 2 | ^12^C_6_H_3_^79^Br_2_O  ^12^C_6_H_3_^79^Br^81^BrO  ^12^C_6_H_3_^81^Br_2_O  ^12^C_7_H_6_^79^Br^81^BrO | 2,4-DiBP |
| 2  (20.0) | 24.23 | 340 (200)  342 (200)  344 (200)  502 (100) | M-CO_2_CH_2_^79^Br^81^Br  M-CO_2_CH_2_^79^Br^81^Br +2  M-CO_2_CH_2_^79^Br^81^Br +4  M-CO_2_CH_2_ +2 | ^12^C_13_H_10_^79^Br_2_O  ^12^C_13_H_10_^79^Br^81^BrO  ^12^C_13_H_10_^81^Br_2_O  ^12^C_13_H_10_^79^Br_2_^81^Br_2_O_2_ | 6-ECFO-BDE-47 |
|  | 27.58 | 341 (200)  354 (100)  356 (200)  358 (100)  560 (100) | M - CO_2_CH_3_  ^79^Br^81^Br + 2  M-CO_2_^79^Br^81^Br  M-CO_2_^79^Br^81^Br +2  M-CO_2_^79^Br^81^Br +4  M + 4 | ^12^C_13_H_9_^79^Br^81^BrO  ^12^C_14_H_12_^79^Br_2_O  ^12^C_14_H_12_^79^Br^81^BrO  ^12^C_14_H_12_^81^Br_2_O  ^12^C_15_H_12_^79^Br_2_^81^Br_2_O_3_ | 3-ECFO-BDE-47 |

Table S4. Percent amino acid sequence identities of two Japanese medaka C1r C1s like sequence with reported fish othologues

|  | ENSORLT00000015464^a^ (ENSORLP00000015463)^b^ | ENSORLT00000015483^a^ (ENSORLP00000015482)^b^ |
| --- | --- | --- |
| carp C1rs-a^a^ | 47.60% | 37.70% |
| carp C1rs-b^b^ | 47.10% | 37.60% |
| trout C1r/C1s^c^ | 58.10% | 32.80% |
| yellow perch C1r/s^d^ | 62.90% | 33.50% |

a:transcript ID in Ensemble Database

b:protein ID in Ensemble Database


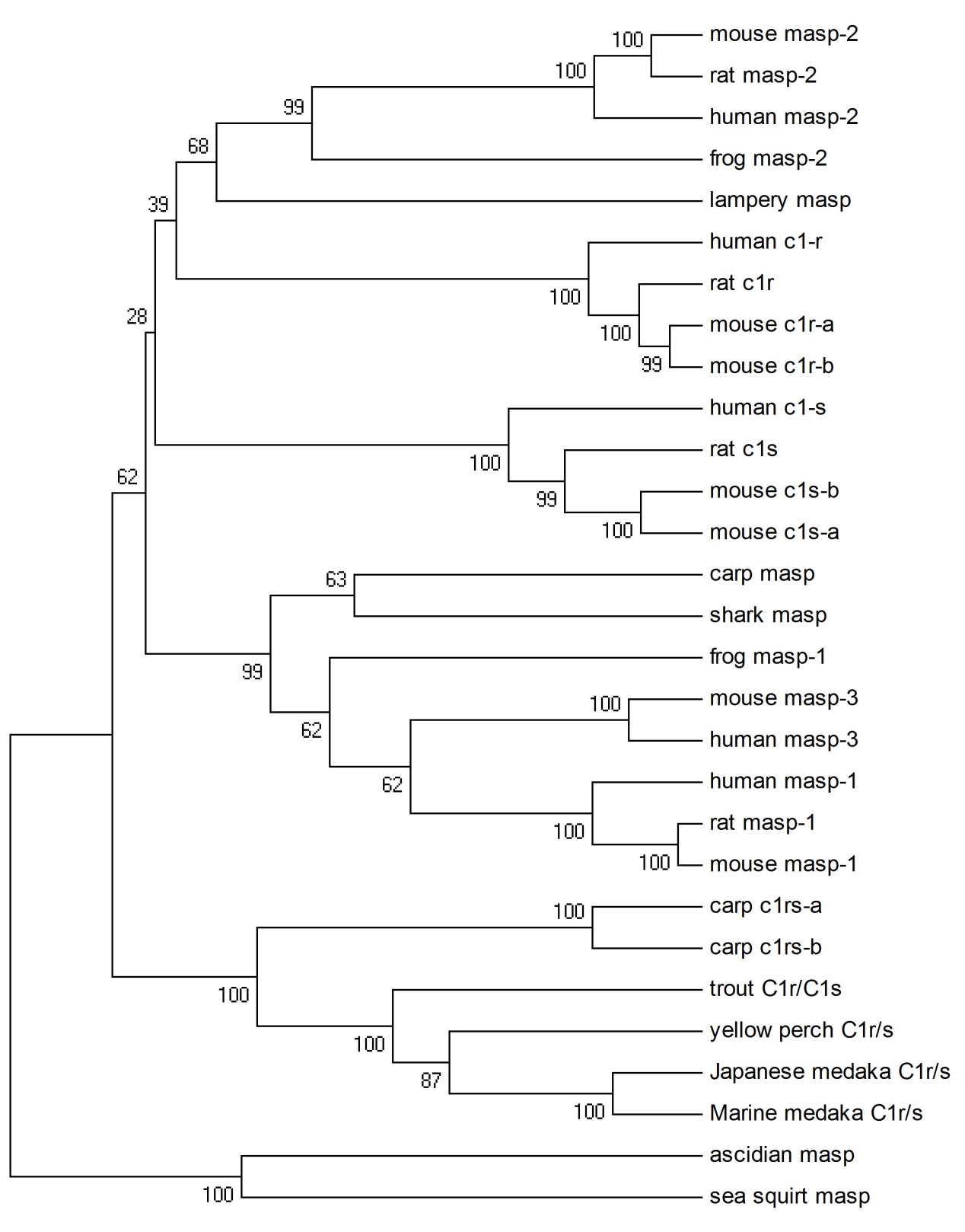


Figure S1. Phylogenetic tree of C1r, C1s and Maspothologuesin multiple species. The tree is constructed by using MUSCLE for multiple sequence alignment and UPGMA method. Bootstrap percentages that support each portioning are given.
